# Digital biomarkers: Convergence of digital health technologies and biomarkers

**DOI:** 10.1038/s41746-022-00583-z

**Published:** 2022-03-25

**Authors:** Srikanth Vasudevan, Anindita Saha, Michelle E. Tarver, Bakul Patel

**Affiliations:** 1 Division of All Hazards Response, Science, and Strategic Partnerships, Office of Strategic Partnerships and Technology Innovation, Center for Devices and Radiological Health, U.S. Food and Drug Administration, Silver Spring, MD USA; 2 Digital Health Center of Excellence, Office of Strategic Partnerships and Technology Innovation, Center for Devices and Radiological Health, U.S. Food and Drug Administration, Silver Spring, MD USA; 3 Office of Strategic Partnerships and Technology Innovation, Center for Devices and Radiological Health, U.S. Food and Drug Administration, Silver Spring, MD USA

**Keywords:** Biomarkers, Health care, Medical research

## Abstract

Increasing digitization across the healthcare continuum has revolutionized medical research, diagnostics, and therapeutics. This digitization has led to rapid advancements in the development and adoption of Digital Health Technologies (DHT) by the healthcare ecosystem. With the proliferation of DHTs, the term ‘digital biomarker’ has been increasingly used to describe a broad array of measurements. Our objectives are to align the meaning of ‘digital biomarker’ with established biomarker terminology and to highlight opportunities to enable consistency in evidence generation and evaluation, improving the assessment of scientific evidence for future digital biomarkers.

## Introduction

Over the last decade, increasing digitization across the healthcare continuum has revolutionized medical research, diagnostics, and therapeutics. This digitization has led to rapid advancements in the development and adoption of digital health technologies (DHT) by consumers, researchers, and providers to enable collection of health-related data outside the traditional clinical setting (Box 1). With the shift to digitization in healthcare, the term ‘digital biomarker’ has been increasingly used to describe a broad array of measurements. There are currently multiple definitions of the term digital biomarker reported in the scientific literature, and some seem to conflate established definitions of a biomarker and a clinical outcomes assessment (COA). Biomarkers and clinical outcome assessments measure different concepts and both could be useful in understanding the impact of a condition on patients. For example, an investigational product used to treat patients with heart failure could be assessed by measuring a biomarker of the heart’s output (left ventricular ejection fraction) as well as through a COA, a subjective measure of how the patient feels (the Kansas City Cardiomyopathy Questionnaire). Conflating the terms can hamper communication and evidence expectations between medical product developers and regulators. Therefore, a clear definition of the term digital biomarker could potentially facilitate the effective use of a DHT in the evaluation of a medical product, potentially increasing patient access to safe and effective medical products. Additionally, with recent advancements in digitization across healthcare, the ability to detect non-biological external factors (e.g., environmental features like pollen count) provides an opportunity to identify predictors and influences on health, that will require systematic development of scientific evidence in the future. Therefore, our objectives are (1) to align the meaning of ‘digital biomarker’ with established terminology on biomarker that will enable consistency in evidence generation and evaluation; and (2) to highlight opportunities that improve the assessment of scientific evidence for future digital biomarkers.

Box 1 Definition of digital health technologies (DHT) A system that uses computing platforms, connectivity, software, and sensors for healthcare and related uses^1^. These technologies span a wide range of uses, from applications to support general wellness to medical device applications such as apps that provide a reminder to stay out of the sun to limit UV exposure. They include technologies intended for use as a medical product such as digital therapeutic that provides cognitive behavior therapy, a medical product that uses consumer technology in fitting a hearing aid, or as an adjunct to other medical products (i.e., devices, drugs, and biologics) such as an app to boost adherence to a therapy. They may also be used to develop or study medical products.

## Definition of a digital biomarker

As defined in the Biomarkers, EndpointS and other Tools (BEST) glossary developed by U.S. Food and Drug Administration (FDA) and National Institutes of Health Biomarker Working Group, a biomarker is “a defined characteristic that is measured as an indicator of normal biological processes, pathogenic processes, or biological responses to an exposure or intervention, including therapeutic interventions”^1^ (e.g., blood pressure). In line with this definition and in a guidance document^2^, FDA defines a digital biomarker to be a characteristic or set of characteristics, collected from digital health technologies, that is measured as an indicator of normal biological processes, pathogenic processes, or responses to an exposure or intervention, including therapeutic interventions. The use of ‘characteristic or set of characteristics’ in the definition of digital biomarkers stems from the ability to derive one or more biomarkers from one or more DHTs simultaneously. In some instances, the characteristics of the host and disease or medical condition can be simultaneously collected and consolidated from multiple DHTs to derive a biomarker. This ability to derive biomarkers from multiple DHTs can potentially provide additional context to enrich normal values for the population, patient-specific baseline values, and assess changes in health status relevant for healthcare applications.

## Examples of digital biomarkers from published literature and a hypothetical case

A number of DHT applications have been published in the peer-reviewed literature. These examples shown in Table 1 are shared to highlight how the biomarker is conceptualized or used. The examples highlighted in this paper may or may not have been substantiated with adequate evidence to validate its use in regulatory submissions. To further clarify the distinctions between digital biomarkers and different types of COAs, Table 2 highlights features of the different types of measures to assess an individual’s functioning by measuring or monitoring their ability to tap on a smart phone screen.

**Table 1:** Examples of digital biomarkers from published literature.

Biomarker category	BEST definition	Digital biomarker example
Diagnostic biomarker	A biomarker used to detect or confirm the presence of a disease or condition of interest or to identify individuals with a subtype of the disease^1^	An algorithmic classification of cardiovascular features extracted from optical sensors on wearable devices to identify atrial fibrillation^5^
Pharmaco-dynamic/response biomarker	A biomarker used to show that a biological response has occurred in an individual who has been exposed to a medical product or an environmental agent^1^	A wrist-worn DHT may collect accelerometer data and use the data to detect physiological changes (for e.g., tremor and bradykinesia) in response to a pharmacological agent. Mahadevan et al. studied its utility in assessing the response to levodopa in patients with Parkinson’s disease^6^
Monitoring biomarker	A biomarker measured repeatedly for assessing the status of a disease or medical condition or for evidence of exposure to (or effect of) a medical product or an environmental agent^1^	An ccelerometer-based sensor device that collects data about chest and limb movement to measure gait in patients with Huntington’s Disease^7^

**Table 2:** Hypothetical example of assessing hand function using a smart phone^a^.

Concept being measured	Type of measure
Tasking a study participant to complete a structured tapping exercise on the smart phone for measuring location of the tap and time delays between taps for identifying signal for an early sign of a neurological disorder	Digital biomarker
Tasking a study participant to complete a structured tapping exercise on the smart phone for measuring the study participant’s functional ability	COA – Performance outcome
Physical function questionnaire that asks a study participant about hand-related activities of daily living	COA – Patient-reported outcome
Clinician observing a study participant complete a hand exercise and grading the participant’s performance	COA – Clinician-reported outcome
Life partner reporting observations of spouse doing certain hand-related functions	COA – Observer-reported outcome

^a^These are theoretical examples. The authors do not assert that hand tapping would be a measure of hand function/dexterity without appropriate evaluation of the analytical and clinical evidence.

## Conclusion

In recent years, the growing confidence in DHTs has led to an increase in the adoption of these technologies by consumers, researchers, and providers to help better understand healthcare outside of the conventional clinical setting. Innovations in DHTs have been led by both traditional (academic and industry) and non-traditional (consumer electronics) manufacturers, with the intent to advance the future of healthcare. This rapid advancement in healthcare enabled by DHTs has made it possible to collect continuous health data from a user’s natural environment, which was once limited by the need to visit a clinical facility. Additionally, this clarification of the meaning of digital biomarkers is consistent with the biomarker definition in the BEST glossary and is used by FDA. While it is clear that the continued development, access, and adoption of digital biomarkers depend on the entire healthcare ecosystem working together, consistent use of the definition of digital biomarker described here will help improve communication critical for medical product development. In addition to biomarkers, it is important to recognize the influence of other external factors (e.g., environment), on a person’s health^3,4^. Scientifically validated external factors with clinical associations to a person’s health, such as local pollen count for asthmatic patients or ultraviolet index for photosensitive individuals, integrated with digital biomarkers may provide greater insights on triggers for diseases and conditions, and could potentially inform more timely prevention, diagnosis, and treatment.

### Reporting summary

Further information on research design is available in the Nature Research Reporting Summary linked to this article.

## Supplementary information

Reporting Summary Checklist

## References

[1] FDA-NIH Biomarker Working Group. *BEST (Biomarkers, EndpointS, and Other Tools) Resource* (FDA-NIH Biomarker Working Group, 2016). 27010052

[2] U.S. Food and Drug Administration. Patient-Focused Drug Development: Collecting Comprehensive and Representative Input. *Final guidance document* https://www.fda.gov/media/139088/download (2020).

[3] van den Brink, W. et al. Digital resilience biomarkers for personalized health maintenance and disease prevention. *Front. Digital Health*10.3389/fdgth.2020.614670 (2021). 10.3389/fdgth.2020.614670 PMC8521930 34713076

[4] Barrett M (2018). AIR Louisville: Addressing asthma with technology, crowdsourcing, cross-sector collaboration, and policy. Health Aff..

[5] Perez MV (2019). Large-scale assessment of a smartwatch to identify atrial fibrillation. N. Engl. J. Med..

[6] Mahadevan N (2020). Development of digital biomarkers for resting tremor and bradykinesia using a wrist-worn wearable device. NPJ Digit. Med..

[7] Andrzejewski KL (2016). Wearable sensors in Huntington disease: A pilot study. J. Huntingt. Dis..

